# Postpartum modern contraceptive use and associated factors in Hossana town

**DOI:** 10.1371/journal.pone.0217167

**Published:** 2019-05-22

**Authors:** Negeso Gebeyehu Gejo, Abebe Alemu Anshebo, Leta Hinkosa Dinsa

**Affiliations:** 1 Department of Midwifery, College of Medicine and Health Sciences, Wachemo University, Hosanna, Ethiopia; 2 Department of Midwifery, College of Medicine and Health Sciences, Wollega University, Nekemte Ethiopia; Anglia Ruskin University, UNITED KINGDOM

## Abstract

**Background:**

Postpartum family planning is the initiation and use of family planning services within the first 12 months following childbirth to impede closely spaced and unintended pregnancies. Globally, spacing pregnancies at least 2 years apart can prevent an estimated 10% of infant deaths and 21% of deaths in children 1–4 years of age. The main purpose of this study was to determine postpartum modern contraceptive use and associated factors in Hossana town, Hadiya zone, Southern nation nationalities peoples region, Ethiopia.

**Methods:**

Facility based cross-sectional study was conducted from June 03 to July 03, 2018, in Hossana town, Hadiya zone. Data was collected by structured questionnaires using face-to-face interview on 368 women. Systematic random sampling technique was employed to approach the study participants. SPSS version 20 software was used for data analysis. Both bivariate and multiple variable logistic regression analysis were computed. Odds ratio with their 95% confidence intervals were calculated and statistical significance was decided if p < 0.05.

**Result:**

Two hundred seventy one (72.9%) women used postpartum modern contraception. Educational status of mothers [AOR = 0.26; 95% Cl; 0.09–0.744], resumption of sex [AOR = 4.20; 95% Cl; 1.533–11.517], menses resumption [AOR = 8.48; 95% Cl; 3.072–23.228] and duration postpartum period [AOR = 0.26; 95% Cl; 0.107–0.644] had significant association with postpartum modern contraceptive use.

**Conclusions:**

The prevalence of postpartum modern contraception use is relatively high. Educational status of mothers, resumption of sex, menses resumption and duration of postpartum period were factors significantly associated with postpartum modern contraceptive use. Improving women education & delivering messages for couples on the risk of getting pregnant prior to menses is crucial.

## Introduction

Postpartum family planning (PPFP) is preclusion of closely spaced and unplanned pregnancies through the use of family planning services within the first 12 months following childbirth [1]. Modern contraceptive method is defined as a product or medical procedure that interferes with the reproduction from acts of sexual intercourse (sterilization male/female, intrauterine devices and systems, subdermal implants, oral contraceptives, male/female condoms, injectables, Diaphragms and cervical caps etc.) [2].

Even though women become fecund before menses resumption, numerous women don’t initiate contraception until their menses reappears. This will increase the risk of getting unintended pregnancy if sex resumed [3, 4].Worldwide, 95% of 1^st^ year postpartum women want to delay another pregnancy at least two years or avoid future pregnancy [5].

Sixty nine percent of women living in sub-Saharan Africa, South Central Asia and Southeast Asia have unmet need for modern contraception. Annually, in these three regions, 49 million women have unintended pregnancies, leading to 21 million unplanned births, 21 million induced abortions (15 million of which are unsafe), 116,000 maternal deaths and the loss of 15 million healthy years of women’s lives [6].

World Health Organization (WHO) technical committee recommends an interval of at least 24 months before couples attempt to become pregnant [1]. A birth-to-pregnancy interval of less than 12 month are more likely to end in potentially unsafe induced abortion, spontaneous abortion, postpartum bleeding and anemia, and are at increased risk for stillbirth, preterm birth, low birth weight, and small size for gestational age. Closely spaced births are also increases the likelihood of chronic undernourishment, stunted growth, and infant mortality [7, 8, 9, 10]. Contraceptive use during the postpartum period significantly reduces rates of maternal and infant mortality by preventing unplanned and unwanted pregnancies and by spacing new pregnancies at least two years after the previous birth [11].

Among women at one month postpartum, the unweighted average modern contraceptive was 8%, ranging from 0% in Burkina Faso to the 44% in Zimbabwe. By the 12^th^ month postpartum, unweighted average increases to 30%, ranging from 6% in Benin to 72% in Indonesia. In Ethiopia, 18% of women use modern contraception at 12^th^ months postpartum [12].

According to Ethiopian demographic and health survey (EDHS) 2016, median birth interval is 34.5 months; thus, half of non-first births occur within 3 years after the first birth. One in three births (32%) occurs within 24–35 months of previous birth, and one in five births (21%) occur within at least 3 years after the previous birth. In Ethiopia, the pregnancy related mortality ratio was 412 maternal deaths per 100,000 live births, the under-5 mortality rate is 67 deaths per 1,000 live births, and the infant mortality rate is 48 deaths per 1,000 live births [13].

Consequently, it is very important to initiate contraception in the postpartum period to avert the tragic maternal and child morbidity and mortality. Studies regarding postpartum contraceptive use and associated factors are limited in Ethiopia, especially in the study area. Thus, this study aims to investigate postpartum modern contraceptive use and associated factors in Hossana town to enhance its practice.

## Materials and methods

A community based cross-sectional study design was conducted from June 03 to July 03, 2018 in Hossana town, capital of Hadiya zone, southern nation nationalities peoples region, Ethiopia. The town has 8 kebeles (lowest administrative units).

The source populations encompassed all reproductive age women, who gave birth in the last 12 months prior to this study. The study population included selected reproductive age women who gave birth in the last 12 months prior to this study.

Sample size was computed using single population proportion formula by taking; 95% confidence level, 5% margin of error, and prevalence of postpartum modern contraceptive use which is 48% (taken from a research conducted in Axum town) [14]. After using correction formula (since the total population is less than 10,000) and adjusting 5% for non-response rate, the minimum sample size was calculated to be 368.

Out of eight kebeles in Hossana town, three kebeles were selected by simple random sampling technique (lottery method). The total sample size for respective kebeles was allocated using proportional allocation by size. Before the actual data collection, a census was done to identify postpartum women in each kebele. The study participants were selected by through systematic random sampling technique. The sampling interval was obtained by dividing the number of postpartum women in each kebele by the proportionally allocated sample for each Kebele. The sampling interval was calculated to be two. Thus, every second postpartum woman was included until the computed sample size for each Kebele is achieved. The first postpartum woman was selected by lottery method.

The questionnaire was first prepared in English, translated to Amharic, and then translated back to English to check for consistency. Its consistency was checked by translating it back to English by two different individuals. Data was collected by Amharic language which is the local language. Data was collected by pretested and structured questionnaire through face to face interview. Data was collected by 3 data collectors and 1 supervisor. Training was given to data collectors and supervisors on the purpose of the study, questionnaire and how to fill responses. The questionnaire was adapted from related published articles by considering objective of the study and local situation [14, 15, 16]. We have assured the validity of the instrument by properly applying validity criteria’s. These are ‘homogeneity’, ‘convergence’ and ‘predictive value’ Reliability was assured by stability (the instrument was given to the same participants more than once under similar circumstances and it was consistent).

Information were collected on socio -demographic data; reproductive history, maternal health care and current practice regarding postpartum contraception; knowledge on PPFP; past experiences with modern contraception service and sexuality related variables. The validity and reliability of the instrument was assured through scientific manner. The questionnaire was pre-tested on 5% (19) of the calculated sample size in Shurmo dubancho kebele which is out of the study area before the actual data collection period. Questionnaire was checked daily to ensure completeness and consistency. Eligibility criterion was all women who gave birth in the last 12 months prior to the study period and reside in Hossana town.

### Measurement

*Knowledge on modern contraception* was measured by using eight questions. Those participants who scored ≥4 had 'good knowledge' and <4 had ‘poor knowledge' [17].

### Data analysis

The collected data was checked manually for its completeness. Then, it was cleaned, coded and entered in to Epi-data 3.1 and exported to statistical package for social science (SPSS) version 20.0 for analysis. Descriptive statistics were done and presented with tables.

Both bivariate and multiple variable logistic regression analyses were used to determine association of independent variables with the dependent variable. At first, bivariate logistic regression analysis was carried out to see association of each independent variable with the dependent variable. Those variables with p < 0.25 at bivariate logistic regression were taken in to multiple variable logistic regression model. Odds ratio with their 95% confidence intervals were calculated and statistical significance was declared if p < 0.05. The Hosmer-Lemeshow statistic had chi-square value of 7.979 and a significance of 0.436 and therefore the model is fit. Multi-collinearity was checked for interaction between independent variables by VIF (Variance inflation factor) which is less than 5.

### Ethics approval and consent to participate

Ethical approval was taken from Wachemo University research and community service vice president office. Formal letters was obtained from the Hossana town health office. Concerned body from respective kebeles officially communicated before commencing the data collection. Informed written consent was obtained from participants prior to engaging to the study. For participants who were unable to write, a right thumbprint was taken as a signature. Parental or legal guardian consent was taken for respondents who were under 18 years of age. Respondents were notified about their right to refuse or terminate at any point of the interview. The information provided by each respondent was kept confidential and de-identified and de-linked and kept in a secure location.

## Result

### Socio-demographic characteristics

A total of 368 women participated in the study with the response rate of 100%. Almost half of the mothers 182 (49.5%) were between 25–29 years of age. The mean age of mothers were 29.12 (SD ± 5.042). 357 (97.0%) were married, 226 (61.4%) were Hadiya and 230 (62.5%) were protestant. 108 (29.3%) mothers can read and write and 12 (3.3%) had no formal education. Regarding mothers’ occupation, 164 (44.6%) were house wives. Majority of respondents 261 (70.9%) earn a monthly income ≥1001 Ethiopian birr (> = 36.75USD) (Table 1).

**Table 1:** Socio-demographic characteristics of women in postpartum period in Hossana town, Southern Ethiopia, 2018 (n = 368).

Variables	Category	Frequency	%
Age	15–19	19	5.2
	20–24	25	6.8
	25–29	182	49.5
	30–34	81	22.0
	> = 35	61	16.6
Marital status	Married	357	97
	Single	4	1.1
	Divorced	7	1.9
Ethnicity	Hadiya	226	61.4
	Kambata	62	16.8
	Amhara	40	10.9
	Oromo	5	1.4
	Silte	30	8.2
	Wolayita	5	1.4
Religion	Orthodox	75	20.4
	Muslim	35	9.5
	Protestant	230	62.5
	Catholic	18	4.9
	Adventist	10	2.7
Educational status	No formal education	12	3.3
	Read and write	108	29.3
	Primary education	75	20.4
	Secondary education	61	16.6
	Diploma & above	112	30.4
Monthly income	<18.36USD*	38	10.3
	18.39–36.71USD	69	18.8
	> = 36.75USD	261	70.9

*1ETB = 0.03671USD

### Fertility and reproductive characteristics

The mean parity of mothers was 2.76 (SD ±1.591) and mean living children was 2.72 (SD ± 1.593). The median birth interval was 18 months. 43 (11.7%) did not intend to have more children in the future. More than three-fourth of mothers, 287 (78.0%) said their menses had resumed after birth. Nearly half of the participants, 178 (48.4%) had jointly decided with their husband to use family planning. More than three-fourth of mothers, 280 (76.1%) had ANC four times. Almost all mothers, 367 (99.7) had delivered at health institution and post natal care. Majority of respondents, 352 (95.7%) had received family counseling during ANC and PNC (Table 2).

**Table 2:** Fertility and reproductive characteristics of women in postpartum period in Hossana town, Southern Ethiopia, 2018 (n = 368*)*.

Variables	Category	Frequency	%
Parity	1–2	38	10.3
	3–4	69	18.8
	≥5	261	70.9
Number of children	1–2	261	54.6
	3–4	126	34.2
	5+	41	11.1
Birth interval (in months)	<24	288	78.3
	24–47	52	14.1
	>48	28	7.6
Reproductive intention	Want to space	248	67.4
	Want to limit	43	11.7
	Undecided	71	19.3
	Want to have a child	6	1.6
Who decide to use family planning	Mainly respondents	33	9.0
	Mainly the husband	157	42.7
	Jointly decision	178	48.4
Postpartum period	<6 months	98	26.6
	≥6months	270	73.4
Did you have ANC during last pregnancy	Yes	357	97.0
	No	11	3.0
If yes how many times?	2–3	78	21.2
	4+	280	76.1
Post natal care	Yes	367	99.7
	No	1	0.3
Place of delivery	Home	1	0.3
	Health institution	367	99.7
Family planning counseling during prenatal and PNC	Yes	352	95.7
	No	16	4.3

### Knowledge on postpartum modern contraception use

Almost all of mothers, 363 (98.6%) had ever heard one method of modern contraception. Nearly half of respondents, 180 (48.9%) mentioned injectable contraception and implants. Majority of the participants, 332 (90.2%) got information about contraceptive methods from health professionals. Majority of the mothers, 349 (94.8%) said the appropriate contraception during postpartum period is modern contraceptive. Regarding the overall knowledge on PPFP, majority of respondents, 355 (96.5%) were knowledgeable.

### Modern contraceptive use in the postpartum period

Modern contraceptive use was 272 (73.9%). The most commonly used method was injectable contraception 152 (48.53%), followed by implants 90 (33.09%) and IUCD 25 (9.19%). Slightly more than one-third, 132 (35.9%) respondents started to use contraception after their menses resumed and 52 (14.2%) at 6 weeks. Majority of the participants, 232 (63.0%) used contraception for spacing and 21 (5.7%) for limiting (Table 3).

**Table 3:** Modern contraceptive use of women in postpartum period in Hossana town, Southern Ethiopia, 2018 (n = 368).

Variable	Category	Frequency	%
Currently using any method of contraception	Yes	272	73.9
	No	96	26.1
If YES, which method(s) are you currently using?	Progestin Only Pill	3	1.1
	Intrauterine Contraceptive Device	25	9.19
	Injectable contraception (DMPA)	152	55.88
	Implants	90	33.09
	Male condom	2	0.74
Why are you using contraception currently?	Want to space	232	85.29
	Want to limit	21	7.72
	Want to have a child	19	6.98
When did you start to use contraception?	Immediately at birth	37	13.6
	Before menses	6	2.2
	After menses	132	48.53
	6 weeks	52	19.12
	10 weeks	45	16.55

Major reason cited for not using contraception is menses not resumed 31 (32.29%) followed by spousal not present 20 (20.8%) (Data is not shown in the table).

### Factors associated with postpartum modern contraceptive use

But in multivariable logistic regression analysis; educational status of the mother, resumed sexual activity, menses resumed and postpartum period were significantly associated (p-value ≤ 0.05) with postpartum family planning use (Table 4).

**Table 4:** Binary and multivariate logistic regression result for factors associated with postpartum family planning use, in Hossana town, Southern Ethiopia, 2018.

Variables	Category	Postpartum family planning use		COR(95% Cl)	AOR (95%Cl)
		Yes Freq. (%)	No Freq. (%)		
Educational status	No formal education	10 (40)	15 (60)	0.133 (0.051–0.346)*	0.016 (0.001–0.223)**
	Read and write	82 (75.9)	26 (24.1)	0.631(0.319–1.248)*	0.874 (0.297–2.578)
	Primary education	58 (75.68)	16 (21.62)	0.725 (0.339–1.550)	0.579 (0.090–0.744)
	Secondary education	37 (62.71)	22 (37.29)	0.336 (0.160–0.706)*	0.259 (0.090–0.744)**
	Diploma & above	85 (83.33)	17 (16.67)	1	1
Monthly income	<18.36USD	18 (47.37)	20 (52.63)	0.269 (0.134–0.540)*	4.157 (0.877–19.714)
	18.39–36.71USD	53 (76.81)	16 (23.19)	0.989 (0.527–1.855)	2.076 (0.634–6.798)
	> = 36.75USD	201 (77.01)	60 (22.99)	1	1
Resumed sexual activity	Yes	258 (85.43)	44 (14.57)	21.779 (11.132–42.611)*	4.201 (1.533–11.517)**
	No	14 (21.21)	52 (78.78)	1	1
Menses resumed	Yes	243 (84.67)	44 (15.33)	9.903 (5.678–17.274)*	8.477 (3.072–23.228)**
	No	29 (35.80)	52 (64.20)	1	1
Currently breast feeding	Yes	248 (77.50)	72 (22.50)	3.444 (1.846–6.427)*	2.789 (0.994–7.825)
	No	24 (50)	24 (50)		
Problem experienced while using contraception	Yes	91 (85.85)	15 (14.15)	1.379 (0.723–2.628)*	0.680 (0.280–1.655)
	No	176 (81.48)	40 (18.52)	1	1
Parity	1–2	135 (67.16)	66 (32.84)	0.538 (0.266–1.215)*	0.296 (0.073–1.206)
	3–4	101 (83.47)	20 (16.53)	1.403 (0.600–3.279)	0.792 (0.207–3.027)
	≥5	36 (78.26)	10 (21.74)	1	1
ANC	Yes	264 (76.30)	82 (23.70)	5.634 (2.283–13.904)*	0.749 (0.034–16.648)
	No	8 (36.36)	14 (63.64)	1	1
Family planning counseling during ANC & PNC	Yes	251 (75.83)	80 (24.17)	2.390 (1.190–4.801)*	4.777 (0.192–118.589)
	No	21 (56.76)	16 (43.24)	1	1
Postpartum period	<6 months	55 (54.46)	46 (45.54)	0.257 (0.156–0.424)	0.263 (0.107–0.644)**
	≥6 months	220 (81.48)	50 (18.52)	1	1

* = P-Value ≤0.25

** = P-Value ≤0.05

Mothers’ educational status showed statistically significant association with postpartum family planning use. Mothers who had no formal education had 98.4% reduced odds to utilize postpartum contraception than those who had diploma and more [AOR = 0.016; 95% Cl; 0.001–0.223], and mothers who had secondary education had 74% reduced odds to use PPFP than their counterparts [AOR = 0.26; 95% Cl; 0.09–0.744].

Resumed sexual activity was the other variable that showed significant association with postpartum family planning use. The odds of using modern contraceptives in women who had resumed sexual activity were 4.20 times higher than in those who had not resumed sexual activity since birth [AOR = 4.20; 95% Cl; 1.533–11.517].

Mothers whose menses returned had odds 8.48 times higher to use postpartum modern contraceptive than their counter parts [AOR = 8.48; 95% Cl; 3.072–23.228].

Duration in postpartum period was found to have a significant association with the use of PPFP. Those mothers whose birth interval was < 6months had 74% reduced odds to utilize postpartum family planning than their counterpart [AOR = 0.26; 95% Cl; 0.107–0.644].

## Discussion

This study assessed the prevalence of postpartum modern contraceptive use and associated factors in Hossana town. In this study, the prevalence of postpartum modern use is 73.9%. The finding of this study is comparable with study done in Kufue, Zambia (73.5%) [18], and slightly lower than study done in Addis Ababa (80.3) [16]. This similarity might be due to the resemblance in socio-demographic characteristics. For instance, in Addis Ababa study, 93.2% of women were married whereas 97% were married in our case. The educational status was almost similar with the above studies.

But, the finding of this study is much higher than study done in India (13.8%) [19], Uganda (28%) [20], Gonder (48.4%) [21], Axum (48%) [14] and Dabat (10.3%) [15].

The discrepancy might be due to variation in the characteristics of the study population. Literatures show that educational status has strong relationship with PPFP use [14, 20, 22].

Consequently, the difference might due to difference in educational status of the mothers. For instance, in the Gonder study, the proportion of women who had no formal education is 21.9% [21] which is much higher than this study (3.3%). Similarly, the proportion of women who had no formal education in Dabat district is 64.4% [15] which is extremely higher than this study (3.3%).

The PPFP utilization in this study is much lower than a study done in Zanjan, Iran [23]. This might be due to difference in the socio-demographic characteristics, time gap of this study conducted and variation in reproductive characteristics.

In this study, overall knowledge on PPFP is 96.5%. The finding of this study is comparable with the study done in Axum (95.6%) [14].

Mothers’ educational status showed statistically significant association with postpartum family planning use. This might be due to the fact that educated women have better understanding of benefits of contraception and risks of short interval pregnancies. They also have better inclination to visit health institution and get the service than those who had no formal education. This finding is consistent with the study done in Axum & Uganda [14, 20].

Resumed sexual activity is the other variable that showed statistically significant association with postpartum family planning use. This might be due to the fact that women who resumed sexual activity have a fear of getting pregnant. Consequently, they seek contraception than those who had not resumed sex. This finding is consistent with study done in Axum [14].

Menses resumption is also showed statistically significant association with postpartum family planning. This might be due to perception of the mothers that once menses has returned the risk of pregnancy is increased and this prompts them to use contraceptive methods. This finding is in line with the studies done in Axum, Addis Ababa and Gonder [14, 16, 21].

Duration in postpartum period was found to have a significant association with the use of PPFP. This might be due to the fact as the duration of postpartum period increases women engage in sexual activity and more over their menses will be resumed. Hence, women might fear of getting pregnant and which boosts them to use contraception.

The finding of this study is in line with study done in Addis Ababa and Gonder which revealed that duration in months after delivery have a significant association with the use of postpartum modern contraception [16, 21].

The limitation of this study includes; the present study concentrated on mothers only but better to incorporate institutions delivering service, health care providers and male partners to identify factors influencing utilization of postpartum modern contraception.

## Conclusions

This study found a relatively high prevalence of postpartum modern contraception use. Educational status of mothers, resumption of sex, menses resumption and duration of postpartum period were factors significantly associated with postpartum modern contraceptive use.

Improving women education in collaboration with different stake holders as it is so central in improving access to postpartum family planning use. Delivering adequate public messages for couples on pregnancy risks prior to the return of menses and encouraging them to use family planning. Conveying messages for couples on having unprotected sexual intercourse put them at the risk of getting pregnant early in postpartum period and boosting them to start postpartum family planning as early as possible.

## Supporting information

S1 File Old PPFP spss doc. (SAV)

S1 Appendix Annexes. (DOCX)

## Abbreviations

AOR: Adjusted odds ratio
ANC: Antenatal Care
COR: Crude odds ratio
EDHS: Ethiopian Demographic and Health Survey
FP: Family planning
IUD: Intrauterine Device
PPFP: Postpartum Family Planning
SD: Standard deviation
SPSS: Statistical Package for Social Science
WHO: World Health Organization
