# Supplementary material for: Postpartum modern contraceptive use and associated factors in Hossana town
Source: PLoS One. 2019 May 22;14(5):e0217167. doi: 10.1371/journal.pone.0217167 (PMC6530850; doi:10.1371/journal.pone.0217167)
Supplement: S1 Appendix — (DOCX) [file pone.0217167.s002.docx]

# Annexes

**Consent Form**

**Consent to participate in study**:

Hello, my name is …………………………, we came from Wachemo University, College of Medicine and Health sciences and doing research on postpartum modern contraceptive use and associated factors among mothers who have children less than 1 year in Hossana town. The aim of the study is to determine prevalence of postpartum modern contraceptive use and associated factors among mothers who have children less than 1 year in Hossana town. If you agree to participate in this study, you will be required to answer a series of question that have been prepared for the study through interview in order to obtain the intended information. All information that will be collected kept in private and will be used only for this study. The form will not bear your name but identification number. Participating in this study is completely voluntary. You have a right not to participate in this study and even if you have already accept to participated in the study you can quit at any time if you feel so. Refusal to participate or withdrawal from the study will not involve penalty or loss of any benefits.

Now, do you agree to participate in the study?

Yes__________ No ____________

If, no respect the decision and thank her. If yes continue the interview.

**Result**

Completed----------------------

Respondent not available-------------------

Refused------------------------------

Partially completed---------------------

Other (please specify) -------------------

Name of the interviewer ……………………. Signature………. Date……………..

Name of the supervisor ……………………. Signature………. Date……………..

**Questionnaire English version**

**Wachemo University, College of Medicine and Health sciences**

Date of interview ___/__/ 2018 Questionnaire code......................

**SECTION 1: SOCIO – DEMOGRAPHIC INFORMATION (circle the correct answer/fill in space provided)**

| **S.no** | \| **Demographic Questions** \| \| --- \| | **Responses** | **Skip** | **Code** |
| --- | --- | --- | --- | --- | --- |
|  | How old are you? (Age in years) | ------------------------------ |  |  |
|  | Marital status | 1. Married 2. Single 3. Divorced 4. Widow |  |  |
|  | Ethnicity | 1. Hadiya 2. Kembeta 3. Amhara 4. Oromo 5. Silte 6. Others (specify)……... |  |  |
|  | Religion | 1. Orthodox 2. Muslim 3. Protestant 4. Catholic 5. Others (specify)……... |  |  |
|  | Educational Status of Mothers | 1. No formal education 2. Read and write 3. Primary education 4. Secondary education 5. Diploma and more |  |  |
|  | Educational Status of Husband | 1. No formal education 2. Read and write 3. Primary education 4. Secondary education 5. Diploma and more |  |  |
|  | Mother’s occupation | 1. Government employee 2. Merchant 3. Farmer 4. House wife 5. Daily laborer 6. Others (specify)……....... |  |  |
|  | Husband’s occupation | 1. Government employee 2. Merchant 3. Farmer 4. Daily laborer 5. Others (specify)……....... |  |  |
|  | Monthly income | --------------------------- Birr |  |  |

**SECTION 2: Reproductive and maternal health service use-related characteristics (circle the correct answer/fill in space provided)**

| **S.no** | \| **Demographic Questions** \| \| --- \| | **Responses** | **Skip** | **Code** |
| --- | --- | --- | --- | --- | --- |
|  | Parity | ------------------------------ |  |  |
|  | Living children | ------------------------------ |  |  |
|  | Birth interval (months) | ------------------------------ |  |  |
|  | Reproductive intention | 1. Want to space 2. Want to limit 3. Undecided 4. Want to have a child |  |  |
|  | Who decide to use family planning | 1. Mainly respondents 2. Mainly the husband 3. Jointly decision |  |  |
|  | Did you have ANC during last pregnancy | 1. Yes 2. No | If no go to Q18 |  |
|  | If yes how many times? | ------------------------------ |  |  |
|  | PNC | 1. Yes 2. No |  |  |
|  | Place of delivery | 1. Home 2. Health institution |  |  |
|  | Postpartum period (wk.) | --------------------------- |  |  |
|  | Family planning counseling during prenatal and PNC | 1. Yes 2. No |  |  |
|  | Menses returned after birth | 1. Yes 2. No |  |  |
|  | Resumed sexual activities by the time of survey | 1. Yes 2. No |  |  |
|  | Currently breast feeding | 1. Yes 2. No |  |  |

**SECTION 3: Knowledge on postpartum modern contraception use (circle the correct answer/fill in space provided)**

| **S.no** | \| **Demographic Questions** \| \| --- \| | **Responses** | **Skip** | **Code** |
| --- | --- | --- | --- | --- | --- |
|  | Have you ever heard any modern method of contraception? | 1. Yes 2. No | If no go to Q28 |  |
|  | If YES, which method(s) do you know? | 1. Progestin Only Pill 2. Combined Oral Contraceptive pill 3. Intrauterine Contraceptive Device 4. Injectable contraception (DMPA) 5. Implants 6. Male condoms 7. Female condoms 8. Sterilization 9. Other (specify------------- |  |  |
|  | Source of information | 1. Health professional 2. TV 3. Radio 4. Friends 5. From pamphlets/ booklet/posters 6. Other (specify)------------- |  |  |
|  | After birth of a child, can a woman become pregnant before her menstrual period has returned? | 1. Yes 2. No 3. I don’t know |  |  |
|  | Short interpregnancy interval has many complications | 1. Yes 2. No 3. I don’t know |  |  |
|  | Postpartum contraception reduces child mortality and improves maternal health. | 1. Yes 2. No 3. I don’t know |  |  |
|  | Where did you get contraceptive methods? | 1. Public health facilities 2. Private clinic 3. I don’t know |  |  |
|  | Which one is suitable contraception during postpartum? | 1. Natural method 2. Modern method 3. I don’t know |  |  |

**SECTION 4: Modern contraceptive use in the postpartum period (circle the correct answer/fill in space provided)**

| **S.no** | \| **Demographic Questions** \| \| --- \| | **Responses** | **Skip** | **Code** |
| --- | --- | --- | --- | --- | --- |
|  | Have you ever used any modern method of contraception | 1. Yes 2. No |  |  |
|  | Have you experience any problem while using contraception? | 1. Yes 2. No |  |  |
|  | Are you currently using any method of contraception? | 1. Yes 2. No | If no go to Q40 |  |
|  | If YES, which method(s) are you currently using? | 1. Progestin Only Pill 2. Combined Oral Contraceptive pill 3. Intrauterine Contraceptive Device 4. Injectable contraception (DMPA) 5. Implants 6. Male condoms 7. Female condoms 8. Sterilization 9. Other (specify------------- |  |  |
|  | Why are you using contraception currently? | 1. Want to space 2. Want to limit 3. Want to have a child 4. Other (specify) ---------------- |  |  |
|  | When did you start to use contraception? | 1. Immediately at birth 2. Before menses 3. After menses 4. 6 weeks 5. 10weeks 6. Other (specify) ---------------- |  |  |
|  | For how long have you been using contraception without discounting it? | ------------------------------ |  |  |
|  | Where did you get the contraception | 1. Public health facilities 2. Private clinic 3. Other (specify) ---------------- |  |  |
|  | If No why? | 1. Religious prohibition 2. Spousal not present 3. Not resume sexual 4. Spousal disapproval 5. Single and had no partner 6. Fear of side effect 7. Not resumed menses/ 8. Other (specify)---------------- |  |  |
|  | If No do you have intention to use in the future? | 1. Yes 2. No |  |  |
|  | If yes for what purpose you will use it? | 1. Want to space 2. Want to limit 3. Want to have a child 4. Other (specify) ---------------- |  |  |
